# Structures of bacterial kynurenine formamidase reveal a crowded binuclear zinc catalytic site primed to generate a potent nucleophile

**DOI:** 10.1042/BJ20140511

**Published:** 2014-08-22

**Authors:** Laura Díaz-Sáez, Velupillai Srikannathasan, Martin Zoltner, William N. Hunter

**Affiliations:** *Division of Biological Chemistry and Drug Discovery, College of Life Sciences, University of Dundee, Dow Street, Dundee DD1 5EH, U.K.

**Keywords:** amidase, binuclear metal site, kynurenine formamidase, tryptophan catabolism, X-ray crystallography, zinc enzyme, ACMSD, α-amino-β-carboxymuconate-ε-semialdehyde decarboxylase, *Ba*KynB, *Bacillus anthracis* KynB, *Bc*KynB, *Burkholderia cenocepacia* KynB, CV, column volume, *Pa*KynB, *Pseudomonas aeruginosa* KynB, KFase, kynurenine formamidase, NCS, non-crystallographic symmetry, NFK, *N*-formyl-L-kynurenine, SEC, size-exclusion chromatography, TEV, tobacco etch virus, XANES, X-ray absorption near-edge structure

## Abstract

Tryptophan is an important precursor for chemical entities that ultimately support the biosynthesis of key metabolites. The second stage of tryptophan catabolism is catalysed by kynurenine formamidase, an enzyme that is different between eukaryotes and prokaryotes. In the present study, we characterize the catalytic properties and present the crystal structures of three bacterial kynurenine formamidases. The structures reveal a new amidase protein fold, a highly organized and distinctive binuclear Zn^2+^ catalytic centre in a confined, hydrophobic and relatively rigid active site. The structure of a complex with 2-aminoacetophenone delineates aspects of molecular recognition extending to the observation that the substrate itself may be conformationally restricted to assist binding in the confined space of the active site and for subsequent processing. The cations occupy a crowded environment, and, unlike most Zn^2+^-dependent enzymes, there is little scope to increase co-ordination number during catalysis. We propose that the presence of a bridging water/hydroxide ligand in conjunction with the placement of an active site histidine supports a distinctive amidation mechanism.

## Abstract 2

Catalytic properties and structures of three bacterial kynurenine formamidases are presented. The dimeric enzyme possesses an uncommon fold and crowded binuclear zinc active site. Fluorescence and structure of a complex inform on molecular recognition and a plausible mechanism is proposed.

## INTRODUCTION

Tryptophan catabolism via the kynurenine pathway supports the biosynthesis of NAD^+^, important neuroactive intermediates in eukaryotes, then anthranilate, quinolate and antibiotics in prokaryotes [1–4]. The proteins involved in tryptophan catabolism are in general highly conserved across species, but with the notable exception of the second enzyme in the pathway: kynurenine formamidase. This enzyme catalyses the conversion of *N*-formyl-L-kynurenine (NFK) into formate and L-kynurenine [2].

In eukaryotes, the enzyme is termed kynurenine formamidase (KFase) and displays relatively low sequence conservation, although the structure itself is well maintained. This is evident from studies with *Sacharyomyces cerevisiae* and *Drosophila melanogaster* KFase, which, although they only share 10% sequence identity, are structurally conserved α/α hydrolases [5,6]. The KFase fold is dominated by a core eight-stranded α-sheet decorated on either side with a number of α-helices. This creates a well-defined cavity at one end of the sheet, leading to a catalytic triad consisting of a serine, histidine and aspartate combination. Overall, there are close structural similarities between KFase and carboxylesterases, but also intriguingly to another enzyme involved in kynurenine biology, namely a 5′-pyridoxyl phosphate-dependent kynurenine aminotransferase [5,6].

The chemistry behind an amidation reaction involves either acidic or basic hydrolysis. It is the latter that applies to these metal-free amidases, which exploit the properties of reactive cysteine or serine residues to create covalent acyl-intermediates during catalysis. Cysteine-dependent amidases are exemplified by trypanothione synthetase amidase [7], nicotinamidase [8] and the nitrilases [9], whereas the eukaryotic KFases from *S. cerevisiae* and *D. melanogaster* present examples of the serine-dependent enzymes with a mechanism that correlates with known amidases [5,6]. In KFase, an ordered mechanism results when the catalytic triad positions histidine to activate serine into a nucleophile that attacks the substrate carbonyl carbon. Subsequent engagement of a water molecule then allows for completion of the reaction.

Our attention was drawn to the prokaryotic KFase KynB (EC 3.5.1.9). Analysis of KynB sequences indicated that they are unrelated to KFase and indeed do not match any known amidase or amidohydrolase. Clearly, KynB is different and so, intrigued, we sought to investigate the structure–activity relationships in this distinctive enzyme. We now report the preparation of efficient recombinant expression systems for KynB from three bacterial sources and their purification. The catalytic properties of the enzymes are characterized and tryptophan fluorescence is used to investigate ligand binding. High-resolution crystal structures have been determined that allow us to describe the architecture of the enzyme extending to the details of the active site, to address metal ion identification and to investigate key features of molecular recognition at the catalytic centre. Taken together, our structural data then allow us to propose a plausible and distinctive mechanism for KynB.

## EXPERIMENTAL PROCEDURES

### Expression plasmid preparation

Genes encoding KynB in *Pseudomonas aeruginosa* (*Pa*KynB, UniProt: Q9I234), *Burkholderia cenocepacia* (*Bc*KynB, UniProt: B4E9I9) and *Bacillus anthracis* (*Ba*KynB, UniProt: Q81PP9) were purchased (Genscript) having been codon optimized for expression in *Escherichia coli* K12. *Pa*KynB and *Bc*KynB encoding genes were cloned into the NdeI/XhoI and *Ba*KynB into the NdeI/BamHI site of a modified pET15b vector (Novagen) to create plasmids that produce an N-terminal hexahistidine-tagged (His-tagged) protein and a tobacco etch virus (TEV) protease cleavage site. All constructs were sequenced to check their integrity.

### Expression and protein purification

Gene expression and purification of the proteins started with freshly transformed *E. coli* BL21(DE3)pLysS in the case of *Pa*KynB and *Bc*KynB systems, and *E. coli* BL21(DE3) for *Ba*KynB. Bacteria were cultured at 37°C in 10 ml of LB broth containing 50 μg· ml^−1^ carbenicillin and, for *Pa*KynB and *Bc*KynB cultures, 20 μg· ml^−1^ chloramphenicol was also present. These cultures were used as inoculum for 1 litre of LB/antibiotic mixtures. The bacteria were cultured at 37°C until an attenuance of 0.6–0.8 at *λ*=600 nm was achieved. The temperature was lowered to 16°C in the case of *Pa*KynB and *Bc*KynB, and to 20°C for *Ba*KynB. Gene expression was induced with 1 mM IPTG and cultures were incubated overnight. Cells were then harvested by centrifugation (4000 ***g*** at 4°C for 10 min). The *Pa*KynB and *Bc*KynB cultures were resuspended in buffer A1 (25 mM Tris/HCl, pH 7.5, and 100 mM NaCl), and *Ba*KynB in buffer A2 (20 mM Tris/HCl, pH 7.4, and 200 mM NaCl) with the addition of complete EDTA-free protease inhibitor cocktail tablets from Roche. The cells were disrupted using a sonicator in the case of *Pa*KynB and *Bc*KynB, and a French press for *Ba*KynB, and homogenates were centrifuged at 40000 ***g*** for 30 min at 4°C. The resulting supernatants were passed through a 0.45 μm filter, and samples containing *Pa*KynB and *Bc*KynB were adjusted to contain 5 mM imidazole. Proteins were loaded on a 5-ml HisTrap HP column (GE Healthcare). After a 10-column volume (CV) washing step using 90% buffers A1 or A2 and 10% of buffer B1 (25 mM Tris/HCl, pH 7.5, 100 mM NaCl and 0.5 M imidazole) for *Pa*KynB and *Bc*KynB, or buffer B2 (20 mM Tris–HCl pH 7.4, 200 mM NaCl, 0.8 M imidazole) for *Ba*KynB, the recombinant proteins were eluted applying a linear imidazole gradient (50–250 mM for *Pa*KynB and *Bc*KynB, and 80–400 mM for *Ba*KynB over 20 CV). The eluted proteins were dialysed into buffer A1 or A2 and incubated overnight with 1 mg of His-tagged TEV protease per 20 mg of protein at 4°C, then applied to a HisTrap HP column equilibrated with buffer A1 or A2 to remove the TEV protease, cleaved peptide and non-cleaved material. Initial experiments indicated that the His-tag of *Pa*KynB was not cleaved efficiently by TEV protease, and protein aggregation/precipitation was pronounced. We left the His-tag in place for further experiments with that protein. The enzyme assay (see later) indicated that the His-tagged *Pa*KynB possessed a level of activity comparable with that of other samples. Fractions containing the proteins were collected, concentrated and applied to a size-exclusion chromatography (SEC) column (HR 16/60, Superdex75 prep grade, GE Healthcare, CV=120 ml) equilibrated with buffer C (10 mM NaH_2_PO_4_/Na_2_HPO_4_, pH 7.8, 20 mM NaCl and 0.5 mM Tris/HCl) in the case of *Pa*KynB and *Bc*KynB, and buffer A1 for *Ba*KynB. The SEC columns had been calibrated with molecular mass standards (thyroglobulin, 670 kDa; γ-globulin, 158 kDa; serum albumin, 67 kDa; ovalbumin, 44 kDa; myoglobin, 17 kDa; vitamin B_12_, 1 kDa). Protein purity and molecular weight were assessed by SDS/PAGE and MALDI–TOF-MS analyses performed at the University of Dundee ‘Fingerprints’ Proteomics Facility using an Applied Biosystems Voyager DE-STR spectrometer. Protein quaternary structure was investigated by native PAGE and SEC.

### Protein concentration determination

Protein concentrations were measured by absorbance at 280 nm using the predicted molar absorption coefficients by ExPASy bioinformatics resource portal ProtParam [10]:

$$\begin{array}{ccc} ɛ(Pa\mathrm{KynB}) & = & 28\;210\;M^{-1}\cdot {\mathrm{cm}}^{-1}\\ ɛ(Bc\mathrm{KynB}) & = & 28\;210\;M^{-1}\cdot {\mathrm{cm}}^{-1}\\ ɛ(Ba\mathrm{KynB}) & = & 20\;970\;M^{-1}\cdot {\mathrm{cm}}^{-1} \end{array}$$

### Enzymatic assay and determination of catalytic parameters

A spectrophotometric assay was developed to obtain *K*_m_, *V*_max_ and *k*_cat_ values for KynB. The assay is based on the increase in absorbance due to L-kynurenine formation [11,12] which is detected by measuring the absorbance at 365 nm with a UV-2450 Shimadzu spectrophotometer over a period of 160 s. The reactions were performed in triplicate using 1 ml final volume, at 25°C, using 0.025, 0.15, 0.25, 0.5, 0.7, 1, 1.5, 2 and 3 mM NFK substrate (Dalton Pharma Services), and 500 ng of enzyme (20 nM). The buffer used contained 0.1 M NaH_2_PO_4_/Na_2_HPO_4_, pH 7.4, and 20 μM ZnCl_2_. Calculations of the initial velocity for each substrate concentration were made by fitting a linear equation to the data of the increase in L-kynurenine concentration during 1 min, and data were analysed using the Michaelis–Menten equation (SigmaPlot, Systat Software). L-Kynurenine concentration was calculated using the Beer–Lambert law and ε_365_ of 4220 M^−1^·cm^−1^ [12].

### Crystallization, diffraction and structure determinations

*Pa*KynB and *Bc*KynB were concentrated to 7.5 mg·ml^−1^ in buffer A1 and *Ba*KynB to 4 mg· ml^−1^ in buffer A2 to provide a stock solution for crystallization experiments. The first crystallization trials used commercial screening sets from Molecular Dimensions and Qiagen and were set up in 96-well sitting drop plates with a Phoenix liquid handling system (Rigaku-MSC) using a ratio of 1:1 for protein/reservoir and final volumes of 0.2 and 0.4 μl for every condition. Plates were incubated at room temperature in a Gallery DT plate hotel (Rigaku-MSC). *Pa*KynB crystallization occurred using the reservoir condition 0.1 M Hepes, pH 7.5, 20% (w/v) PEG 4000 and 10% (v/v) propan-2-ol. Crystals grew to a maximum dimension of approximately 0.25 mm, over 5 days. The *Bc*KynB crystal was obtained using a reservoir comprising 10–16% (w/v) PEG 3350, 5 mM CoCl_2_, 5 mM CdCl_2_, 5 mM MgCl_2_ and 5 mM NiCl_2_. Before data collection, the crystals were soaked for a few seconds in the reservoir liquid with added 25% (v/v) glycerol and then flash frozen in liquid nitrogen. Diffraction data for *Pa*KynB and *Bc*KynB were collected using the Diamond Light Source beamline IO3 using a Pilatus 6M-F detector, indexed and integrated using XDS [13], scaled and analysed with SCALA and POINTLESS [14] from the CCP4 suite [15].

The initial crystallization conditions for *Ba*KynB gave only small mechanically twinned samples and optimization was carried out in 24-well hanging drop plates using a ratio of 1:1 in final volumes of 2 and 4 μl at 20°C. *Ba*KynB was incubated with 8 mM L-kynurenine for 10 min at room temperature, and a structure was obtained from a crystal grown in a drop equilibrating with a reservoir containing 100 mM Tris/HCl, pH 8.5, 150 mM MgCl_2_, 30% (w/v) PEG 4000 and 1.5% (v/v) dioxane. The presence of dioxane was crucial to improve the morphology such that single-crystal blocks could be obtained. Crystals of *Ba*KynB were flash frozen directly from the drops in which they grew. Diffraction data were obtained using the in-house X-ray facility (Rigaku M007HF X-ray generator with a Saturn 944HG+ CCD detector). Data were indexed and integrated using iMOSFLM [16] and scaled and analysed by AIMLESS from the CCP4 suite [15]. To obtain crystals of the *Ba*KynB–2-aminoacetophenone complex, protein was incubated with 5% (v/v) 2-aminoacetophenone before the crystallization plate was set up. The ligand was previously dissolved to a final concentration of 20% (v/v) into buffer A2 containing 20% (w/v) PEG 4000. The reservoir contained 140 mM MgCl_2_, 30% (w/v) PEG 4000 and 100 mM Tris/HCl, pH 8.5. Diffraction data for the *Ba*KynB–2-aminoacetophene complex were obtained at the Diamond Light Source using beamline IO3.

The structure of *Pa*KynB was determined by molecular replacement (MOLREP) [17] using a single polypeptide (molecule A) for a putative metal-dependent hydrolase from *Geobacillus stearothermophilus* (PDB code 1R61). All water molecules and ions were removed from the model. The search model, which shares 25% sequence identity with *Pa*KynB, is at 2.5 Å resolution, and although Zn^2+^ is assigned in the possible active site, it is with low occupancy (0.5), with an inflated thermal parameter (*B*-factor approximately 90 Å^2^) and distances to potential co-ordinating groups that are more typical of hydrogen-bonding interactions than metal ion ligand associations. There is no information concerning why Zn^2+^ was assigned here, and there are no publications on this structure. The *Bc*KynB structure was solved using a partially refined *Pa*KynB as the search model (64% sequence identity), and then the *Ba*KynB structure was solved using *Bc*KynB (40% sequence identity) as the search model. Similar protocols were applied in the refinement of all structures. This involved first a rigid body refinement as part of the molecular replacement calculations, then iterative cycles of restrained refinement combining REFMAC5 [18] with electron and difference density map inspections, and model manipulations in COOT [19]. The starting *B*-factors for each model were derived from the Wilson *B*-factor. There are multiple polypeptide chains in the asymmetric units (two subunits for *Pa*KynB and four subunits for *Bc*KynB, *Ba*KynB and the *Ba*KynB–2-aminoacetophenone complex), and tight non-crystallographic symmetry (NCS) restraints were imposed at the onset of refinement, which were gradually released during the process. Although L-kynurenine was present in the crystallization mixture used for *Ba*KynB, there was no evidence for the enzyme product in electron density maps. Even with a much greater amount of 2-aminoacetophenone present, we only observed ordered binding of ligand in one active site. Once the protein models were complete, alternative side-chain conformers, water molecules, metal ions and ligands were incorporated into the models. A correction for a twinning component of 0.15, calculated in REFMAC, was applied in refinement of the *Ba*KynB–2-aminoacetophenone complex. Refinements were terminated when there were no significant changes in *R*_work_ and *R*_free_ values and inspection of the difference density maps suggested that no further corrections or additions were justified.

The presence of Zn^2+^ was first suggested by anomalous difference Fourier maps (not shown) and subsequently confirmed in the *Pa*KynB and *Ba*KynB crystals by X-ray absorption near-edge structure (XANES) spectra measured at Diamond Light Source on beamline IO3 using a Vortex Silicon Drift detector and an excitation energy range from 9626.7 to 9690.14 eV (Supplementary Figure S1 at http://www.biochemj.org/bj/462/bj4620581add.htm). Since no Zn^2+^ was added during protein production, purification or crystallization, then it is most likely to be derived from the media used to culture the *E. coli*.

The presence of Cd^2+^ and Zn^2+^ in the active site of *Bc*KynB was confirmed on the basis of the electron density and anomalous scattering. Positive density in a difference Fourier map calculated for the 12 sulfur atoms in the asymmetric unit gave 1.1 σ per electron. The average *B*-factor for these 12 atoms is 16.4 Å^2^. This was used for the determination of the number of electrons present at the *Bc*KynB metal ion sites. The difference Fourier map revealed peaks with heights of 46.5 and 68.4 σ, respectively. These height values and the difference between them are constant within the four active sites in the asymmetric unit, and indicate the presence of two different metal atoms per active site. Comparing atomic numbers between the different metals present in the crystallization condition, one Cd^2+^ and one Zn^2+^ were added to these positions. The average *B*-factors refined to 11.4 and 10.1 Å^2^, respectively. A *B*-factor-adjusted calculation suggests that one site is occupied by an ion consisting of approximately 28 e^−^ and the other about 48 e^−^. Values of 28 and 46 e^−^ would be expected for Zn^2+^ and Cd^2+^, respectively. An anomalous dispersion difference Fourier calculation provided a further check. At the wavelength used for diffraction measurements, *λ*=0.9795 Å, the theoretical *f*″ values for zinc and cadmium are 2.480 and 2.132 e^−^ [20]. In an anomalous difference Fourier a slightly larger signal should therefore occur at the Zn^2+^. Indeed this is observed in all four active sites of the asymmetric unit. The average heights of the peaks are 24.2 σ at Zn^2+^ and 22.5 σ at Cd^2+^ (Supplementary Figure S2 at http://www.biochemj.org/bj/462/bj4620581add.htm).

MolProbity [21] was used to assess geometry of all models. Secondary-structure determination involved use of DSSP [22] and visual inspection, and subunit interface surface area calculations were carried out with PISA [23]. Figures were prepared using ALINE [24] and PyMOL (http://www.pymol.org). The DALI server [26] was used to search the PDB for structural homologues, whereas superpositions were calculated using DALILITE [27]. Relevant crystallographic statistics and geometric details of the refined models are reported in Table 1.

**Table 1:** Crystallographic statistics

Structure	*Pa*KynB	*Bc*KynB	*Ba*KynB	*Ba*KynB–ligand
PDB code	4COB	4COG	4CO9	4CZ1
Space group	*P*3_1_21	*P*2_1_	*P*2_1_	*P*2_1_
Wavelength (Å)	0.9795	0.9795	1.5418	0.9791
Unit cell dimensions *a*, *b*, *c* (Å)	112.7, 112.7, 90.76	76.86, 50.12, 35.2, β=94.14°	73.17, 66.02, 83.76, β=90.32°	73.69, 66.56, 84.06, β=90.24°
Resolution range* (Å)	28.90–2.37	28.37–1.60	42.48–1.95	42.64–2.25
Number of reflections	133546	479924	202165	104599
Unique reflections	27365	134904	58336	38046
Completeness (%)	99.2 (94.5)	99.3 (99.8)	99.4 (94.1)	98.1 (97.7)
*R*_merge_†	0.057 (0.571)	0.068 (0.477)	0.063 (0.166)	0.164 (0.551)
Redundancy	4.9	3.6	3.5	2.7
<*I*/*σ*(*I*)>	16.8 (2.4)	12.8 (3)	11.1 (4.3)	4.8 (2.2)
Wilson *B* (Å^2^)	43.87	14.97	11.84	15.33
*R*_work_‡/*R*_free_§	0.1519/0.1945	0.1489/0.1842	0.1715/0.2066	0.1837/0.2241
Number of residues/waters/ligands and metals	412/181/6	831/1068/38	829/906/18	825/793/1/11
Diffraction precision indicator∥ (Å)	0.198	0.068	0.137	0.086
Bond lengths (Å)/angles¶ (°)	0.018/1.890	0.025/2.429	0.008/1.322	0.017/1.684
Average *B*-factors (Å^2^)	51.4	16.4	15.3	20.9
Protein atoms	3207	6533	6658	6521
Water molecules	181	1068	906	411
Metal ions	4 Zn^2+^	4 Zn^2+^, 4 Cd^2+^, 8 Mg^2+^	8 Zn^2+^, 5 Mg^2+^	8 Zn^2+^, 3 Mg^2+^
Ligands	12 Glycerol, 10 1,2-ethanediol, 1 PEG	2 Dioxane, 3 1,2-ethanediol	2-Aminoacetophenone	
Ramachandran analyses				
Favoured regions (%)	96.1	96.1	97.2	96.5
Allowed regions (%)	100	100	100	100

*Values in parentheses refer to the highest resolution shell.

†*R*_merge_=Σ*_hkl_Σ_i_|I_i_*(*hkl*) − <I(*hkl*)>*|/*Σ*_hkl_Σ_i_I_i_*(*hkl*); where *I_i_*(*hkl*) is the intensity of the *i*th measurement of reflection *hkl* and <I(*hkl*)> is the mean value of *I_i_*(*hkl*) for all *i* measurements.

‡*R*_work_=Σ*_hkl_*∥*F*_o_|−|*F*_c_∥/Σ|*F*_o_|, where *F*_o_ is the observed structure factor and *F*_c_ is the calculated structure factor.

§*R*_free_ is the same as *R*_work_ except calculated with a subset, 5%, of data that are excluded from the refinement calculations.

∥Diffraction Precision Index [42].

¶[43].

### Fluorescence spectroscopy with *Ba*KynB

Fluorescence measurements for *Ba*KynB were performed using a LS-55 PerkinElmer spectrometer to investigate ligand association/disassociation. To determine the peak of maximum emission due to tryptophan, the excitation wavelength used was 280 nm and the emission wavelength detection ranged from 300 to 400 nm. The sensitivity of the detector was fixed to 800 V, and the peak of maximum emission was experimentally determined at 337 nm.

**Table 2:** Catalytic parameters *V*_max_, maximum catalytic velocity; *K*_m_, Michaelis-Menten constant; specific activity (SA), amount of active enzyme from the total protein quantity; *k*_cat_, number of substrate molecules that are transformed per active site and per time unit. *k*_cat_/*K*_m_ defines the catalytic efficiency.

Enzyme	*V*_max_ (nmol·min^−1^)	*K*_m_ (mM)	*k*_cat_ (s^−1^)	*k*_cat_/*K*_m_ (M^−1^·s^−1^) × 10^4^	SA (μM·min^−1^·mg^−1^)
*Ba*KynB	65.41±2.64	0.40±0.05	50.56	12.64	130.82
*Bc*KynB	58.15±0.98	0.57±0.02	43.94	7.71	116.30
*Pa*KynB-His	147.99±8.42	0.98±0.13	114.21	11.65	295.98

The sample (2 ml) contained 20 μg of *Ba*KynB (400 nM), 0.02 M Tris/HCl, pH 7.4, buffer and 0.2 M NaCl. The two products of the KynB catalysed reaction, L-kynurenine and formate, and 2-aminoacetophenone were investigated separately. Concentrations ranged from 0 to 800 μM in the case of L-kynurenine and 2-aminoacetophenone and from 0 to 500 μM for formate. No fluorescence emission from either L-kynurenine or 2-aminoacetophenone was observed under the experimental conditions. In the presence of increasing concentrations of L-kynurenine and 2-aminoacetophenone, a decrease and shift of the maximum fluorescence intensity was observed. In the case of formate, no variation in the fluorescence intensity was detected. Data are represented as percentage of active site saturation within the different compound concentrations (Supplementary Figures S3 and S4 at http://www.biochemj.org/bj/462/bj4620581add.htm), and disassociation constants were determined.

## RESULTS AND DISCUSSION

### Enzyme purification and quaternary structure

Highly efficient recombinant protein expression systems for the enzymes from *B. anthracis* (*Ba*KynB), *B. cenocepacia* (*Bc*KynB) and *P. aeruginosa* (*Pa*KynB) were prepared. The total protein yields, after purification, from the bacterial cultures were about 10 mg· l^−1^ for *Pa*KynB and *Bc*KynB, and 50 mg· l^−1^ for *Ba*KynB. The theoretical polypeptide mass of KynB is about 23 kDa. During the final chromatography step of purification, i.e. SEC, all three proteins were eluted as a single species with approximate mass of 40 kDa and native gels also identified that a single dimeric species was observed in each case.

### Enzyme assays

The kinetic properties of the enzymes were determined using a spectrophotometric assay and are reported in Table 2. The assays confirmed that active *bone fide* KFases had been purified; for example, in the case of *Ba*KynB, approximate values for *K*_m_ 0.4 μM, *V*_max_ 65 nmol· min^−1^, *k*_cat_ 50 s^−1^ and specific activity 130 μM min^−1^· mg^−1^ were derived. No substrate inhibition was observed under the assay conditions and NFK concentrations that were used. In the case of *Pa*KynB, assays were carried out using protein still carrying the His-tag due to aggregation problems when removal of the tag was attempted. The kinetic parameters for *Pa*KynB, *Ba*KynB and *Bc*KynB are similar to each other. Comparisons of kinetic properties of enzymes is often complicated by the use of different assay conditions and this applies to studies with KynB from *Streptomyces parvulus* [11] and *Ralstonia metallidurans* [1] which were conducted at 30°C and 37°C, respectively. In these cases, the activities are lower than we report with reduced specific activities of about 27 and 4 units· mg^−1^ noted. A more similar room-temperature assay was reported for KynB from *Bacillus cereus* [12] and this gave a specific activity of 68.5 μM min^−1^· mg^−1^ with *k*_cat_/*K*_m_ of 18.3 × 10^4^ M^−1^· s^−1^, values comparable with those from our observations.

### Comments on crystallographic analyses and metal ion identification

The crystal structures of all three enzymes were determined (Table 1). The first analysis, *Pa*KynB at about 2.4 Å resolution identified two cations in the active site, tentatively assigned as a binuclear Zn^2+^ site. A more accurate model was sought, and the structure of *Bc*KynB was determined at 1.6 Å resolution. However, these highly ordered crystals could only by obtained in the presence of different transition metal cations including Co^2+^, Ni^2+^ and Cd^2+^. In addition, glycerol, the optimized cryo-protectant, was bound to the metal ions, blocking the active site, so preventing ligand studies. A structure was required without Cd^2+^ being present, or indeed any transition metal ions other than Zn^2+^ and for which we could obtain data to inform on substrate recognition. Highly ordered structures of *Ba*KynB and a complex with 2-aminoacetophenone at resolutions of 1.9 and 2.3 Å resulted and confirmed the binuclear Zn^2+^ environment. XANES scans confirmed the presence of Zn^2+^ in crystals of *Pa*KynB and in *Ba*KynB (Supplementary Figure S1).

### The overall structure of KynB

**Figure 1:**
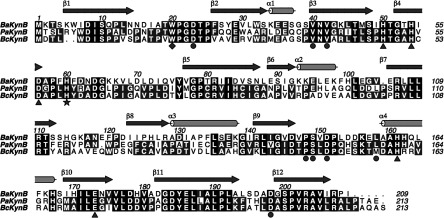
Sequence alignments and assignment of secondary structure for *Ba*KynB Protein sequence alignment from *Ba*KynB, *Pa*KynB and *Bc*KynB. Blue arrows (β-sheets) and red cylinders (α-helixes) indicate the secondary structure of *Ba*KynB. Triangles mark the metal-binding amino acids. Circles mark amino acids in the active site. The star marks a key amino acid for the reaction, His^60^. The diamond marks the tryptophan located at the active site pocket.

The secondary and subunit structure of KynB, NCS of the dimer and detailed architecture of the active site are all highly conserved for the three enzymes. The average NCS values for overlay of Cα atoms range from 0.22 Å, for *Bc*KynB, to 0.32 Å for both *Ba*KynB and *Pa*KynB. The Gram-negative KynB structures are more similar to each other (rmsd overlay of Cα atoms is 0.70 Å) than to the Gram-positive *Ba*KynB (rmsd 1.25 Å in each case). This maps to sequence identities of about 64% for the enzymes from the Gram-negative organisms to 40% when compared with Gram-positive KynB (Figure 1). The structures are, however, so similar, particularly the detail in the active site which is highly conserved (see below), that we primarily detail *Ba*KynB mainly because for that example we also have data that inform on the molecular recognition of ligands in the active site.

**Figure 2:**
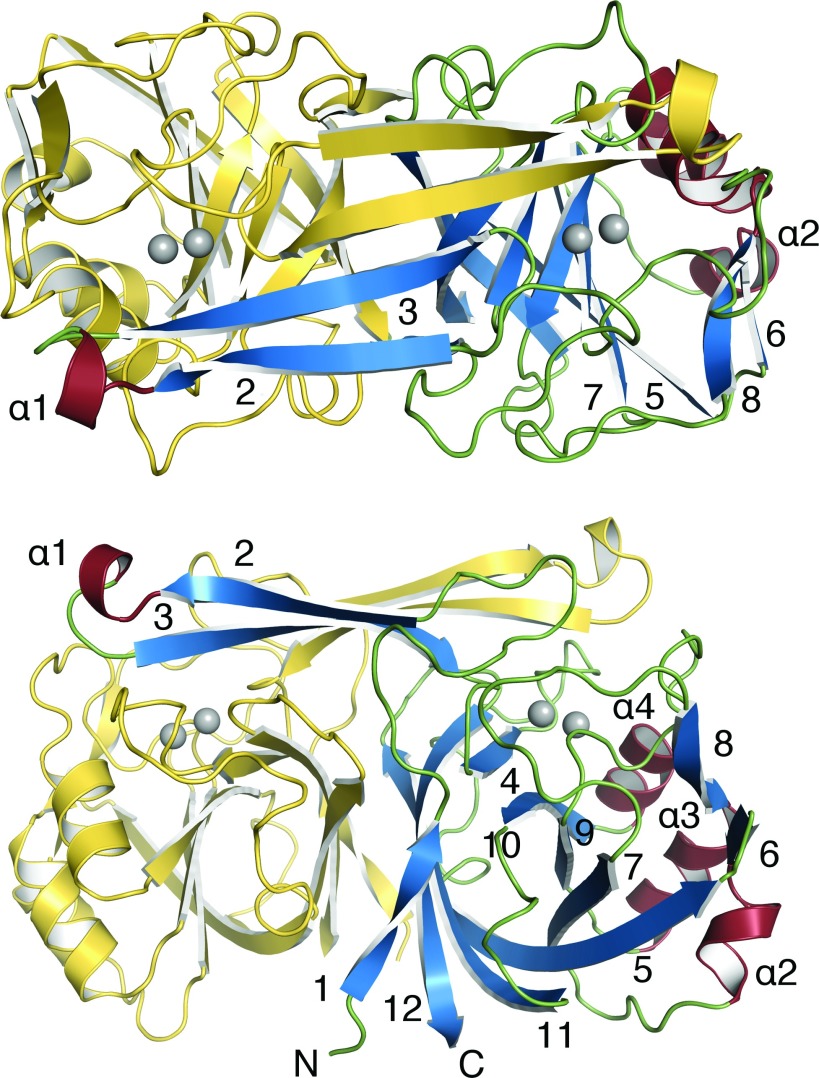
Ribbon diagram of the *Ba*KynB and location of the active site, orthogonal views Zn^2+^ ions are shown as grey spheres. For one subunit, the terminal positions of the polypeptide are labelled N and C, helices are labelled and β-strands numbered. (**A**) Top view, (**B**) side view.

The KynB subunit, approximate mass 23 kDa, comprises just over 200 residues with about 50% in 12 β-strands and 15% in four α-helices (Figures 1 and 2). Three short segments of 3_10_-helix occur between β4 and β5. The molecule gives the appearance of a distorted eight-stranded β-barrel by treating β1 and β4 as a single strand. Three α-helices cluster on one side of the β-barrel, and on the other side is the dimer interface. The fold is not common and a search of the PDB reveals a significant similarity to only three other proteins for which there are little or no published biochemical data. These orthologues are a putative metal-dependent hydrolase/cyclase (PDB codes 3KRV and 1R61, *Z*-scores 27, 26, sequence identity 25%), isatin hydrolase carrying a single Mn^2+^ (PDB code 4J0N, *Z*-score 23, sequence identity 23%) and a hypothetical protein with no metal ion present (PDB code 2B0A, *Z*-score 20, sequence identity 23%).

The KynB dimer (Figure 2) displays comparatively high values, between 22% and 27%, for the accessible surface area of subunits that interact with the partner. This is consistent with the observation of only dimeric species in SEC and native gels. The dimer is stabilized by hydrophobic interactions primarily involving side chains from strands β1, β11 and β12, together with a contribution from short helical segments between residues 68 and 74. The formation of an antiparallel four-stranded β-sheet involving β2–β3 from each subunit is a pronounced feature of the dimer. The N-terminal segment of β3 contributes to formation of the active site, which is mainly created by the partner subunit. In the dimer, the two catalytic sites are separated by about 30 Å.

### The active site of KynB

**Figure 3:**
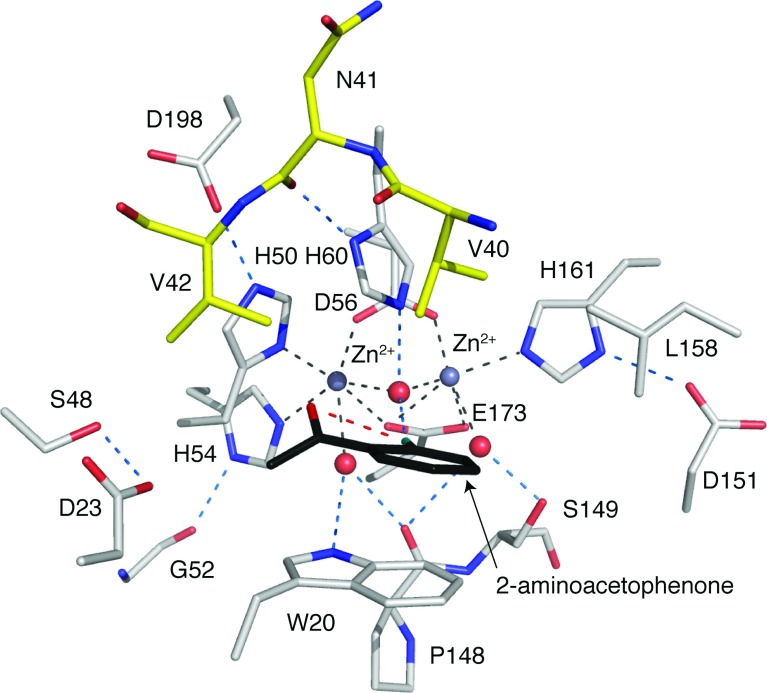
Active site of the *Ba*KynB–2-aminoacetophenone complex Zn^2+^ is a grey sphere, grey broken lines mark co-ordination to amino acid side chains and waters/hydroxide (red spheres). Amino acid atomic positions are coloured with C in grey or yellow depending on subunit, N in blue, O in red, and 2-aminoacetophenone is shown with C in black, N in cyan and O in red. Blue broken lines represent potential hydrogen bonds and a single red broken line indicates the intramolecular interaction in the ligand.

The enzyme active site is a narrow cavity approximately 7 Å×12 Å and 10 Å in depth, lined by hydrophobic walls composed of Trp^20^, His^60^ and Leu^158^ from one subunit, and Val^40^ and Val^42^ from the partner (Figure 3). Four of these residues are strictly conserved in *Bc*KynB and *Pa*KynB, whereas Leu1^58^ corresponds to Met^157^ in *Bc*KynB (Figure 1). At the base of the cavity is a polar floor, where two Zn^2+^ ions, separated by a distance of 3.1 Å, are bound. This separation is unusually close for a binuclear site when compared with other systems [28].

**Figure 4:**
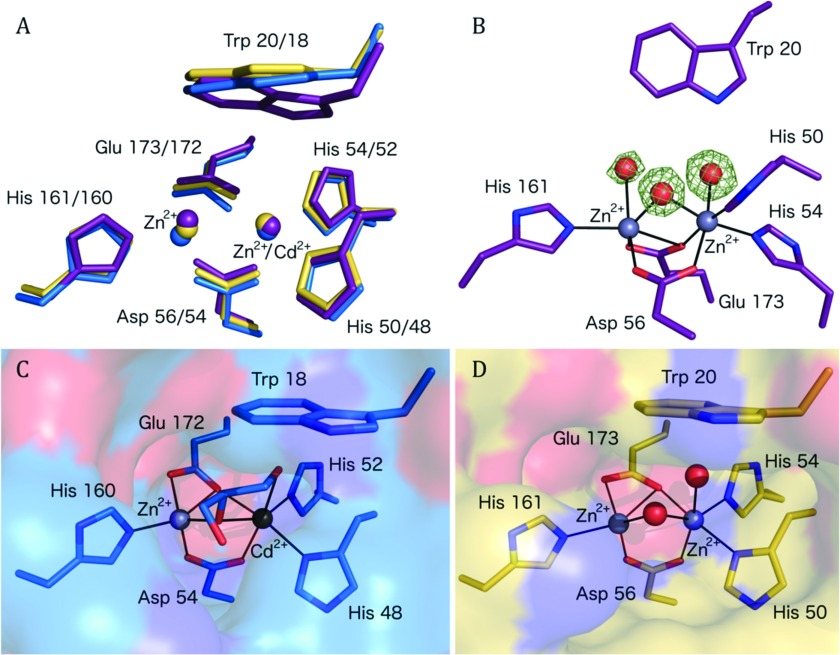
The active site of KynB is highly conserved (**A**) Superimposition of *Ba*KynB (violet), *Bc*KynB (blue) and *Pa*KynB (yellow) active site structures. (**B**) The *Ba*KynB active site with difference density for the three water molecules (red spheres) in the active site depicted as dark green chicken wire and contoured at 5σ. (**C**) The *Bc*KynB active site with glycerol bound to the metal ions. (**D**) The *Pa*KynB active site. Continuous lines represent co-ordinating contacts to the Zn^2+^ ions (grey spheres).

The co-ordination environment at the binuclear Zn^2+^ site is highly organized (Figure 3). One Zn^2+^ is co-ordinated by Asp^56^, His^161^, Glu^173^ and a water molecule/hydroxide ion in a tetrahedral fashion with distances less than 2.3 Å. The water/hydroxide co-ordinates with the other cation and acts as a bridge for the binuclear site. A water molecule and the other oxygen of the side chain of Glu^173^ are about 2.5 Å from the cation, and together with the adjacent metal ion crowd around this Zn^2+^ to provide a distorted octahedral environment. In a similar fashion, the other cation also has four ligands co-ordinating with distances less than 2.3 Å. These are His^50^, His^54^, Asp^56^ and the bridging water/hydroxide. Then, another water and OE1 of Glu^173^ are 2.4 Å distant from this Zn^2+^. The water molecules that co-ordinate the metal ions also interact by hydrogen-bond formation with the enzyme. This involves interactions with the main chain carbonyl of Pro^148^ and NE1 of Trp^20^ in one case and the side chain of Ser^149^ in the other. The bridging water or hydroxide forms a hydrogen bond with NE2 His^60^. Such interactions may help to stabilize the active site configuration. In similar fashion, the three co-ordinating histidine side chains are held in place by hydrogen bonds that link Asp^151^, Asp^198^ with His^161^ and His^50^, respectively, then His54 interacts with the carbonyl group of Gly^52^. All of these residues and their contributions to the organization of the active site are highly conserved in the three types of KynB structures characterized (Figures 1 and 4).

There are 380 unique sequences annotated as a bacterial KFase in UniProt [29]. The list is reduced to 336 entries when filtered for those with ≥30% sequence identity with *Ba*KynB using the NCBI BLAST server [30]. The sequences were aligned (Clustal Omega) [31,32] and the conservation of key active site residues investigated. His^54^, Asp^56^ and His^60^ are strictly conserved in all sequences, whereas His^50^, His^161^ and Glu^173^ are maintained in 99.7% of the entries. Trp^20^, which contributes a significant hydrophobic character to the substrate-binding site, is strictly conserved in 81% of the entries. Conservative substitutions for phenylalanine and tyrosine account for a further 17.8% of the entries, confirming the importance of an aromatic group at this position.

### Tryptophan fluorescence

Having identified Trp^20^ in the active site, we used fluorescence spectroscopy to investigate binding of L-kynurenine and formate, the products of the formamidase reaction. We also investigated 2-aminoacetophenone given chemical similarities to part of L-kynurenine. This ligand provides the aromatic component of substrate and product and primarily lacks only the 2-amino-4-oxobutanoic acid moiety. Formate did not elicit any spectroscopic change but L-kynurenine and 2-aminoacetophenone gave comparable *K*_d_ values of approximately 60 and 50 μM, respectively (Supplementary Figures S3 and S4). A *Ba*KynB–2-aminoacetophenone complex structure then revealed aspects of molecular recognition in the active site (Figure 3 and Supplementary Figure S5 at http://www.biochemj.org/bj/462/bj4620581add.htm). As we will show, this is consistent with the ligands binding in the vicinity of the active site tryptophan with comparable affinity and suggests that 2-aminoacetophenone represents a suitable molecule from which we can derive information on aspects of substrate recognition. Formic acid did not elicit any change in tryptophan fluorescence, suggesting that this moiety of substrate and one product of the KynB-catalysed reaction does not interact with the active site tryptophan.

### Recognition of a conformationally restricted substrate is implied

We were unable to obtain the structure of a complex of KynB with the products of catalysis, formate or kynurenine. However, the characterization of a *Ba*KynB–2-aminoacetophenone complex structure at 2.25 Å resolution provides a suitable template to inform on aspects of molecular recognition in the active site (Figure 3). Key to ligand, and by implication substrate, binding is the interaction with Trp^20^. There are also van der Waals associations with Val^40^ from the partner subunit. The 2-amino group forms a hydrogen bond to the Zn^2+^ bridging water or hydroxide. In this respect, the complex derived may closely mimic the configuration at the completion of the catalytic reaction and immediately prior to the replacement of product by the incoming substrate for another round of catalysis. An intramolecular hydrogen bond between the amine and carbonyl groups of 2-aminoacetophenone appeared to be significant. A 3D search of the Cambridge Structural Database [33] using 2-aminoacetophenone as the template identified 28 similar compounds with structures determined at atomic resolution and all of which display the same interaction. Therefore, such an intramolecular hydrogen bond probably defines or constrains the substrate conformation, and orients the scissile bond such that the formyl moiety would be directed over between His^60^ and Ser^149^. These are two strictly conserved residues in KynB sequences. The complex structure suggests then that when substrate binds the oxygen of the formyl substituent could displace a water that binds Zn^2+^ weakly and perhaps also interact directly with Ser^149^. The orientation or alignment is then fixed for nucleophilic attack to occur at carbon as is described further below.

### Mechanistic considerations and comparisons with metallohydrolases

Although the enzyme fold is different, the binuclear Zn^2+^ catalytic centre of KynB suggests some similarity to mononuclear and binuclear metalloenzymes in the amidohydrolase superfamily. Such enzymes exploit the Lewis acid properties of the cation, decrease the p*K*_a_ of water and generate a hydroxide nucleophile to initiate catalysis [34,35]. They bind and polarize substrate during an intermediate state in the catalytic cycle and generally, then use an acidic glutamate or aspartate to donate a solvent-acquired proton as the intermediate collapses to release products. Examples of such metallohydrolases include type B β-lactamases [28,36], phosphotriesterases [37], leucine aminopeptidases [38] and dihydro-orotases [39]. Constant features of this superfamily [34] include using an acidic component as proton donor and the presence of flexible loops to engage with substrate are in particular noted in the (β/α)_8_ triose phosphate isomerase-type barrel structures. A relevant example actually occurs further down in the kynurenine pathway, it is the enzyme α-amino-β-carboxymuconate-ε-semialdehyde decarboxylase (ACMSD) [40,41]. ACMSD possess a single Zn^2+^ ion co-ordinated by three histidine residues and an aspartate. Other (β/α)_8_ barrel enzymes such as specific phosphodiesterases and dihydro-orotases also possess a carboxylated lysine, which binds two metal ions, typically Zn^2+^, holding them about 3.5–4.0 Å apart and with enough room around the metal ions to allow for a co-ordination increase from four to five or six in the intermediate state of catalysis [34,35].

KynB immediately struck us as distinct with a binuclear Zn^2+^ site, cations only 3.1 Å apart, which is closer than in other cases, in a more crowded environment and with little scope to increase the co-ordination number given that six co-ordinating groups already surround each ion. This suggests then that straightforward replacement of a co-ordinating water ligand might be important. Furthermore, unlike other metallohydrolases where conformational flexibility appears to be important [34,36,37], the catalytic site of KynB is relatively rigid with an average thermal parameter (*B*-factor) of 9.6 Å^2^ compared with an overall average of 15.1 Å^2^ in *Ba*KynB.

**Figure 5:**
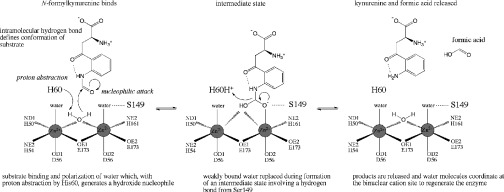
A proposed mechanism for the amidase KynB (*S*)-2-Amino-4-(2-formamidophenyl)-4-oxobutanoic acid (*N*-formylkynurenine) is converted into (*S*)-2-amino-4-(2-aminophenyl)-4-oxobutanoic acid (kynurenine).

Based on the structural data and consistency with the fluorescence measurements, we can now suggest a plausible and distinctive mechanism (Figure 5). The fixed conformation of a planar substrate would assist binding in the small rigid active site cavity. His^60^, a strictly conserved active site residue, in conjunction with the powerful Lewis acid activating binuclear Zn^2+^ environment, is ideally positioned to acquire a proton from the bridging water to generate a potent nucleophilic hydroxide for attack at the carbonyl component of substrate. Direct interaction between the substrate and Zn^2+^ as a catalytic intermediate is formed would probably involve replacement of weakly co-ordinated water assisted by interaction with Ser^149^. The intermediate would collapse as His^60^, which in the complex is 3.6 Å from the 2-amino group of the ligand, donates the proton to form the amine. The C–N bond breaks and products are released.

## Online data

Supplementary data
