# Supplementary material for: Structures of bacterial kynurenine formamidase reveal a crowded binuclear zinc catalytic site primed to generate a potent nucleophile
Source: Biochem J. 2014 Aug 22;462(Pt 3):581–9. doi: 10.1042/BJ20140511 (PMC4243253; doi:10.1042/BJ20140511)
Supplement: Supplementary data [file bj4620581add.pdf]

BJ Biomolecules
Biochem. J. (2014) **462**, 581–589 (Printed in Great Britain) doi:10.1042/BJ20140511
online data

SUPPLEMENTARY ONLINE DATA

Structures of bacterial kynurenine formamidase reveal a crowded binuclear zinc catalytic site primed to generate a potent nucleophile

Laura DÍAZ-SÁEZ*, Velupillai SRIKANNATHASAN*, Martin ZOLTNER* and William N. HUNTER*[1]
*Division of Biological Chemistry and Drug Discovery, College of Life Sciences, University of Dundee, Dow Street, Dundee DD1 5EH, U.K.

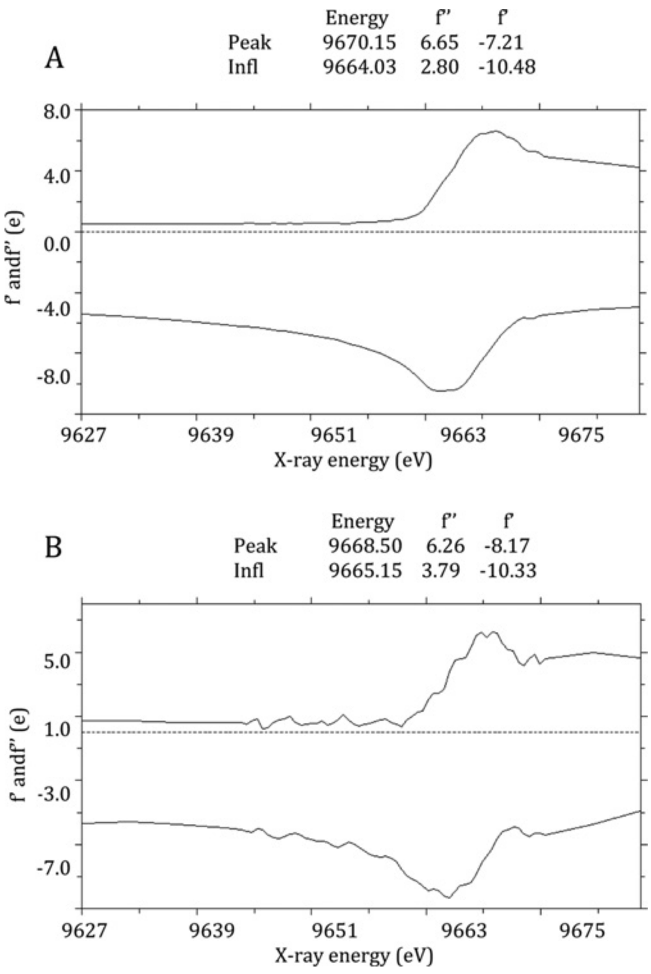

Figure S1 XANES spectra

K-edge XANES spectra from *Ba*KynB (**A**) and *Pa*KynB (**B**) showing the anomalous scattering factor *f*′ and *f*″ values within a range of X-ray energy (from 9626.7 to 9690.14 eV).

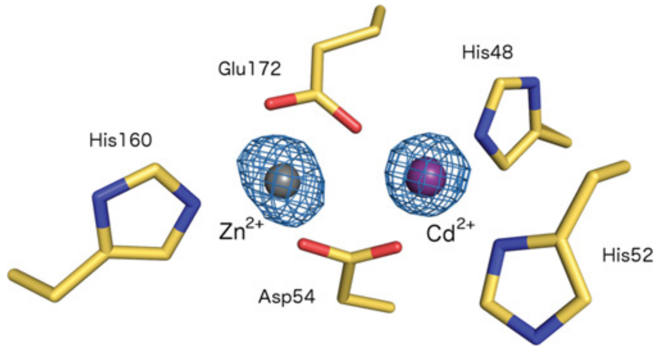

Figure S2 Anomalous difference Fourier map for *Bc*KynB

The map, blue chicken wire, is contoured at 6σ. Zn^{2+} and Cd^{2+} are shown as grey and purple spheres respectively. Water molecules and glycerol have been omitted for the purpose of clarity.

Biochemical Journal www.biochemj.org

[1] To whom correspondence should be addressed (email w.n.hunter@dundee.ac.uk).
Atomic co-ordinates and structure factors have been deposited in the PDB under codes 4COG, 4COB, 4CO9 and 4CZ1.

© 2014 The Author(s)
This is an Open Access article distributed under the terms of the Creative Commons Attribution Licence (CC-BY) (http://creativecommons.org/licenses/by/3.0/) which permits unrestricted use, distribution and reproduction in any medium, provided the original work is properly cited.

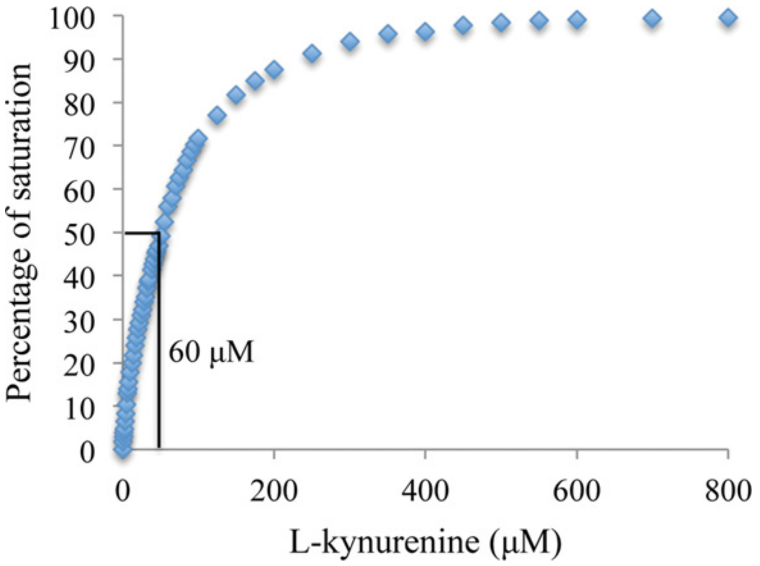

Figure S3 L-Kynurenine binding

Plot derived from fluorescence spectroscopy showing the percentage of active site saturation at different L-kynurenine concentrations.

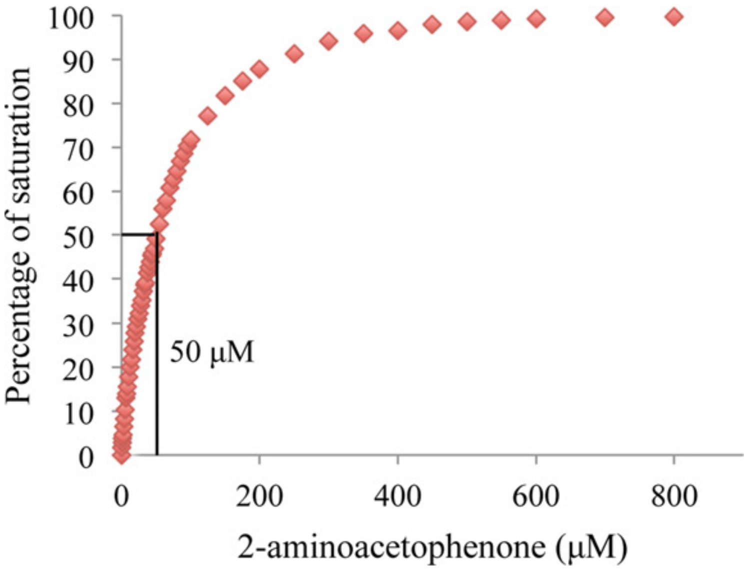

Figure S4 2-Aminoacetophenone binding

Plot derived from fluorescence spectroscopy showing the percentage of active site saturation at different 2-aminoacetophenone concentrations.

© 2014 The Author(s)
This is an Open Access article distributed under the terms of the Creative Commons Attribution Licence (CC-BY) (http://creativecommons.org/licenses/by/3.0/) which permits unrestricted use, distribution and reproduction in any medium, provided the original work is properly cited.

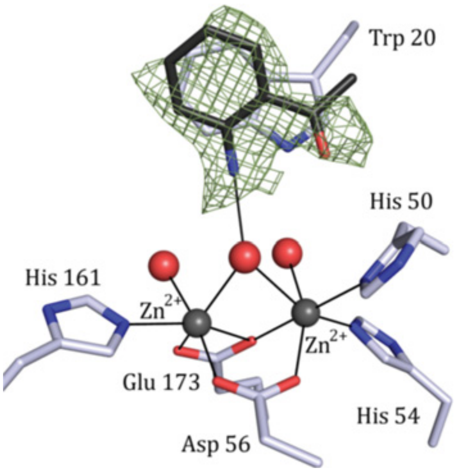

Figure S5 Omit difference density for 2-aminoacetophenone

The $F_o - F_c$ omit map is shown as green chicken wire and contoured at 1.5σ.

Received 17 April 2014/27 May 2014; accepted 19 June 2014
Published as BJ Immediate Publication 19 June 2014, doi:10.1042/BJ20140511

© 2014 The Author(s)
This is an Open Access article distributed under the terms of the Creative Commons Attribution Licence (CC-BY) (http://creativecommons.org/licenses/by/3.0/) which permits unrestricted use, distribution and reproduction in any medium, provided the original work is properly cited.
